# Help-seeking journey to accessing audiology services in a peri-urban community in South Africa

**DOI:** 10.4102/sajcd.v71i1.1002

**Published:** 2024-03-20

**Authors:** Thobekile K. Mtimkulu, Katijah Khoza-Shangase

**Affiliations:** 1 Department of Audiology, Faculty of Human and Community Development, University of the Witwatersrand, Johannesburg, South Africa

**Keywords:** hearing impairment, audiology, help-seeking, access, context, peri-urban, South Africa

## Abstract

**Background:**

Hearing impairment is an invisible disability affecting one in five people globally. Its ability to affect participation in activities of daily living means that it requires prompt identification and intervention.

**Objective:**

This article aims to define the process of accessing audiologists from the onset of symptoms for adults with hearing impairment in a peri-urban community in South Africa.

**Method:**

Twenty-three participants were recruited through purposive sampling from an audiology department of a public hospital. Semi-structured interviews were conducted using an interview guide, and data were mapped according to the participants’ responses from the onset of ear and hearing symptoms to the point of audiologist consultation for analysis.

**Results:**

Seventeen (74%) participants had long journeys to accessing the audiologist after seeking help from multiple providers, with those with short journeys (26%) being referred mostly by public healthcare providers. Despite participants being from one peri-urban community, their journeys were influenced by socio-economics, health illiteracy and other structural factors. Finally, Ear-Nose-Throat specialists linked participants with audiology services.

**Conclusion:**

Accessing audiology services is a complex process in some contexts. The disparities in the social environment, lifestyle factors and pluralistic healthcare models influence access to audiologists. Healthcare providers must take cognisance of the journeys of adults with hearing impairment in their clinical interventions. Universal health coverage, in the form of the planned National Health Insurance (NHI) for all South African citizens, will play an important role in addressing the societal inequalities in accessing healthcare. Factors leading to long journeys should be addressed to facilitate early intervention.

**Contribution:**

The study raises implications for the planned NHI in South Africa, suggesting that universal health coverage could play a vital role in addressing societal inequalities in accessing healthcare, including audiology services.

## Introduction

Hearing impairment is a sensory disability with a mostly insidious onset and progression (Carson, 2005; World Health Organization [WHO], 2023). While it has been ranked as the fifth highest cause of years lived with a disability (The Lancet, 2016), evidence indicates that living with hearing loss has the primary effect of restricting communication, adding psycho-social strain and changing one’s quality of life (Carson, 2005; Cunningham & Tucci, 2017).

Based on these changes, a process of self-assessment imposed by the hearing difficulties influences decisions towards help-seeking (Carson, 2005). Once the hearing difficulties are acknowledged and accepted, audiologists are the ideal healthcare providers to diagnose the hearing impairment and recommend a management plan as this is their scope of practice (Chisolm et al., 2007; McMahon et al., 2013). Typically, audiologists often work together in a pathway with Ear Nose and Throat (ENT) specialists and general practitioners (GPs) as part of a referral network for affected patients (Carson, 2005; Duijvestijn et al., 2003; Laplante-Levesque et al., 2012).

This pathway to the ear and hearing care specialists is easily accessible, affordable and available in high-income countries (HICs) where healthcare services including human resources are well developed. Hearing help-seeking research indicates that participants easily accessed audiologists in countries such as Canada, Germany, Australia and the United States (Carson, 2005; Duijvestijn et al., 2003; Hickson & Scarinci, 2007; Laplante-Levesque et al., 2012). These well-established pathways in HICs are generally short, and the ideal healthcare provider is mostly known by the help seekers. However, the accessibility of audiologists for adults with hearing impairment in other countries cannot be generalised from the sparse studies available from HICs. Unfortunately, a search for similar studies in low- and middle-income countries (LMICs) such as South Africa also yielded a dearth in results.

In LMICs, investigations into pathways for seeking help for other chronic diseases such as breast cancer, rheumatoid arthritis and tuberculosis have revealed different pathways (Moodley et al., 2016; Pati et al., 2019; Pronyk et al., 2001). Help seekers in India and South Africa had either short or long journeys, with some using a combination of multiple providers before accessing the ‘ideal caregiver’. Some journeys involved traditional healers, allopathic providers and religious leaders together with Western medical healthcare. Participants also used public or private healthcare services beginning at the primary healthcare clinic (PHC) level or even at higher levels of healthcare such as hospitals for various reasons. Furthermore, it was also apparent in these studies that healthcare specialists were not the first contact for most of the patients (Mkize & Uys, 2004; Moodley et al., 2016; Pati et al., 2019). Despite this evidence, researchers have argued that the individual is the decisive agent at the centre of this process (Cornally & McCarthy, 2011; MacKian, 2003). However, help-seeking is a complex process that can be influenced by socio-cultural factors such as seeking help from more than one helper and more than one type of help provider in culturally and linguistically diverse contexts (Abaerei et al., 2017; Nadler, 1987; Saunders et al., 2012; Southall et al., 2010).

South Africa is one such context that is culturally and linguistically diverse, with a quadruple burden of disease amplified by unique healthcare system challenges (Pillay-van Wyk et al., 2016). Specific to the challenges is a dual healthcare system – public and private healthcare sectors – that has resulted in an incongruence in demand versus supply (Khoza-Shangase, 2020), as well as capacity versus demand quandaries. Healthcare professional-to-patient ratio in the public healthcare sector is poor resulting in limited or unavailable healthcare services, and this is worse so for professions such as audiology (Khoza-Shangase, 2020; Khoza-Shangase et al., 2017; Pillay et al., 2020). Pillay and colleagues (2020) reported a 0.57 per 10 000 in speech-language and hearing professional to population ratio within the South African context. This finding adds more complexity to the help-seeking journey of the hearing-impaired individual.

Another challenge in this LMIC context is the poor social determinants of health that prevail. The distribution of power, money and other material resources is not equal across the country and even between population groups and socio-economic groupings (Abaerei et al., 2017; Coovadia et al., 2019). The South African Government acknowledges these challenges and has a phased plan to implement universal health coverage via a National Health Insurance (NHI) as part of the National Development Plan 2030 to improve healthcare, socio-economics and other related contextual factors. The main purpose of the NHI is affordable and accessible healthcare for all South Africans (Pauw, 2021), and this should include the availability and approachability of the needed service (Cu et al., 2021; Pauw, 2021). Despite this plan, the current social inequality has created disparities in as far as healthcare access is concerned. Unfortunately, ear and hearing help-seeking research has not exhaustively reported on the influence of the context on this subject.

However, in a South African study, Mkize and Uys (2004) found that participants with a mental illness first sought help primarily from faith or traditional healers, then subsequently from the PHC and district hospital before being referred to a specialised (psychiatric) hospital. Only a few participants were able to be referred directly to mental healthcare services, partly because of the severity of their illness. For most participants in this context, various channels of care were used in search of an ideal caregiver, influenced by socioeconomics, structural environment, health system and even the support of community members. A few other studies from India, Pakistan and Uganda also confirm this pertinent finding (Musinguzi et al., 2018; Pati et al., 2019; Pirani et al., 2015).

Evidently, because of the bio-psycho-social dynamics of each context, help-seeking involves several steps taken by those who are ill in their search for an ideal care provider (Mkize & Uys, 2004). Hence, as part of a bigger study titled ‘In pursuit of preventive audiology: Help-seeking behaviour patterns of adults with hearing impairment in a peri-urban community in South Africa’, the aim of this study was to define the process from the onset of ear and hearing symptoms to accessing audiology services for adults with hearing impairment in a peri-urban community in South Africa.

## Research methods and design

A descriptive qualitative study design on adults with hearing impairment using a non-probability purposive sampling method was adopted (De Vos et al., 2005). Data were collected using in-depth semi-structured interviews using a self-developed interview guide (Appendix 1) in a public hospital with an audiology department. This public hospital is located in a mostly rural province within a district with a population of 212 670 (Statistics South Africa, 2023). This peri-urban area is found on the periphery of an urban and regional centre (Johannesburg) and a rural environment (UNESCO, 2014).

To capture a broad range of experiences, maximum variation sampling was used (Cresswell, 2007) with a set of inclusion and exclusion criteria distinguishing the type of participants required for the study. Potential participants were required to be 18 years old and above, including all genders, have their initial audiology consult in the department and present with any type, degree or severity of hearing loss. Participants were excluded when they were younger than 18 years and had cognitive or linguistic challenges that prevented them from giving consent and participating in the study. The departmental diary of the public hospital was used as a reference for identifying participants. For an informed choice to be made, potential participants who had an audiology consultation on the day were approached, and the purpose of the study was explained including the risks, benefits, affiliation of the researcher and anticipated outcomes. Once participants agreed to participate, formal written informed consent was obtained and the right to withdraw from the study at any point during the interview was assured.

A total of 23 adults representing the socio-demographics of the country were recruited. The intended sample size was 30 participants; however, the sample size was influenced by data saturation. Data saturation was confirmed for a category when no new data emerged from the findings in response to any question. As soon as similar responses were found repeatedly, confidence was reached that data saturation had occurred. Because of the qualitative nature of the study, it was important to remember that the purpose of the study was to understand the phenomenon under study through in-depth, detailed information as opposed to making generalisations through a large data sample (Cresswell, 2007; De Vos et al., 2005).

The researcher audio-recorded the interviews with each participant in a private room, at their convenience, to avoid any biased responses that may have arisen to influence the audiology consultation (Cresswell, 2007; McGrath et al., 2019). Telephonic interviews, where the degree of hearing impairment was not a barrier, were conducted with those participants who were not available at the time of data collection. All adults were over the age of 18 years and were able to answer the questions independently or with the assistance of a family member.

The raw data were then transcribed and translated, where required with the use of Google Translate and the researchers as multilingual speakers of most South African languages into English. Following this process, the researcher familiarised herself with the data through multiple readings (Bengtsson, 2016). The transcribed data were analysed according to the following categories: demographics, onset of symptoms and audiometry results. The responses to the journeys were mapped from the first provider of care to the point of reaching the audiologist at the current setting (Mkize & Uys, 2004). An independent person also transcribed the data independently as a measure of truthfulness and to increase the reliability of the data (Sutton & Austin, 2015; Vaismoradi et al., 2013). Any disagreements between the researcher and the independent person were discussed and resolved in line with the objectives of the study.

A pilot study with three participants was conducted prior to the main study to test the nature of the questions for veracity, credibility and suitability to ensure quality interviewing during the main investigation (De Vos et al., 2005).

All precautions and regulations were followed pertaining to data collection during the time of the coronavirus disease 2019 (COVID-19) pandemic, as the study was conducted at the height of the pandemic in the country (Department of Cooperative Governance, 2021; WHO, 2022).

### Ethical considerations

Ethical clearance to conduct this study was obtained from the University of Witwatersrand, Human Research Ethics Committee (No. M201003) on 08 March 2021 as well as the North West Provincial Department of Health prior to commencing with interviews. The World Medical Association (WMA) Declaration of Helsinki (2013) guided the ethical considerations for this study.

## Results

Of the 23 participants in this study, 17 (73.9%) were classified as older adults while 6 (26%) were below the age of 60 years. An older adult is defined as the stage of retirement where an adult was previously employed and or may receive an older person’s social grant from the state (SA Government, 2022). Therefore, with the average age of the sample being 67.8 years, most participants were retired and not economically active. The study revealed that out of eight participants who were in the adult stage of being employable (15–64 years; Statistics South Africa, 2023), only two were working at the time of data collection. The audiological profile of the participants indicated participants generally presenting with a moderate hearing impairment (49.6 dBHL, s.d. ± 9.1), calculated as a four-frequency pure tone average (4FPTA) in the better ear (WHO, 2023).

The results of this study revealed that journeys to accessing audiology services comprised short and long journeys. Most participants (74%) navigated multiple social networks to reach audiology services with only 26% of the participants reaching the healthcare provider through a self-referral or shortly after one referral.

Table 1 shows the short journeys as described by some of the participants in their help-seeking process. Participants were promptly able to access audiology services from the onset of their help-seeking journey through using mostly healthcare providers.

‘I was at the clinic. I saw the doctor, and that doctor then phoned here at [*name of hospital*] to come …’ (Participant 22, 86 years, male, pensioner)

**TABLE 1:** Participants’ short journeys of help-seeking (*n* = 6).

Participant	Short journeys
Participant 5	Public hospital audiologist
Participant 19	Public hospital audiologist
Participant 12	Private healthcare worker → Public hospital audiologist
Participant 21	Public hospital ENT specialist → Public hospital audiologist
Participant 22	Public healthcare clinic → Public hospital audiologist
Participant 23	Neighbour → Public hospital audiologist

*Source:* Mtimkulu, T.K. (2022). In pursuit of preventive audiology: Help-seeking behaviour patterns of adults with hearing impairment in a peri-urban community in South Africa, Unpublished MA dissertation, Wits University, Johannesburg, South Africa

The ease of access to the audiologist by the participants through first-hand knowledge of the services made the process shorter. Those who used non-healthcare networks stated the following:

‘As I said, I went to my neighbour for help.’ (Participant 23, 64 years, female, unemployed)

As indicated, most participants used public rather than private healthcare services, regardless of their occupational status. The researchers posit that Participant 12 used private medical care because of an existing relationship they had with the medical doctor. This is possibly because they had consulted the private medical doctor previously for their general ailments considering their age (82 years).

Table 2 depicts the long process of seeking help from the onset of symptoms until participants reached audiology services. Most participants (74%) interacted with a variety of healthcare workers from the private as well as the public healthcare sector at all levels of care (primary, secondary, tertiary). Informal social networks were also part of the help-seeking process. Thus, participants had unique pathways as they made their way through the healthcare system from one healthcare professional to another seemingly without a solution:

‘She went to the chemist, and they only syringed it. He syringed the ear, but that was not it … It was really hearing struggle.’ (Participant 4, 82 years, female, pensioner)

**TABLE 2:** Participants’ long journeys of help-seeking (*n* = 17).

Participant	Long journeys-
Participant 2	Private doctor → Family friend (HCW) → Public hospital audiologist
Participant 7	Public hospital endocrine specialist → Public hospital ENT specialist → Public hospital audiologist
Participant 9	Friend → Family (HCW) → Public hospital audiologist
Participant 11	Hearing centre → OTC → Friend → Public hospital audiologist
Participant 13	Public healthcare clinic → Public healthcare clinic → Public hospital ENT specialist → Public hospital audiologist
Participant 14	Public healthcare clinic → Public hospital ENT specialist → Public hospital audiologist
Participant 15	Public healthcare clinic → Public healthcare clinic → Public hospital ENT specialist → Public hospital audiologist
Participant 17	Employer HCW → Public hospital ENT specialist → Public hospital audiologist
Participant 1	Hearing centre → Private audiologist → Public tertiary hospital → Public hospital audiologist (2)
Participant 3	Public healthcare clinic → Public hospital ENT specialist → Family friend (HCW) → Public hospital audiologist
Participant 4	Chemist → Private doctor → Acquaintance/Colleague → Public hospital audiologist
Participant 16	Traditional medicine → Public healthcare clinic → Public hospital ENT specialist → Public healthcare audiologist
Participant 6	Private doctor → Public audiologist → Public doctor → Public hospital ENT specialist → Public hospital audiologist
Participant 8	Friend (HCW) → Private ENT specialist → Private audiologist → Private ENT Public hospital ENT specialist → Public hospital audiologist
Participant 10	Private doctor → Private ENT specialist → (Family) Healthcare worker in hospital → Public hospital Audiologist
Participant 18	Public hospital ENT specialist → Private doctor → Public hospital ENT specialist → Public hospital audiologist
Participant 20	Public healthcare clinic → Public hospital ENT specialist → Public healthcare clinic → Public hospital ENT specialist → Public healthcare clinic → Public hospital ENT specialist → COVID-19 vaccination site → Public hospital audiologist

*Source:* Mtimkulu, T.K. (2022). In pursuit of preventive audiology: Help-seeking behaviour patterns of adults with hearing impairment in a peri-urban community in South Africa, Unpublished MA dissertation, Wits University, Johannesburg, South Africa

HCW, healthcare workers; OTC, over-the-counter; ENT, ear nose and throat; COVID-19, coronavirus disease 2019.

Participant 20 reflected on his long journey with the following words:

‘Ya, that was for the third time that I went to the clinic [*pauses*], the nurse gave me a referral letter but the third time I went there, the doctor [*at the clinic*] was there, and he was able to help me.’ (Participant 20, 56 years, female, unemployed)

Interestingly, some participants returned to the same healthcare worker whom they had consulted earlier in their journeys:

‘I quickly went to [*specialist’s name*]. He also checked me thoroughly, then he referred me to the private audiologist. Then she referred me back to [*specialist’s name*]. So, [*specialist’s name*] realised that the money she was asking for is very high - over 27 or 24 thousand. So, he said he would refer me to the hospital [*public ENT specialist*], then the hospital will refer me to the audiology.’ (Participant 8, 64 years, male, unemployed)

Public healthcare clinics and ENT specialists also contributed to the long journeys because of various contextual reasons. In between, participants also consulted private healthcare services in pursuit of a possible quick solution:

‘I went to a private doctor. I was looking for help… but an assault at the taxi rank made me go back to the specialist. [*public ENT specialist*].’ (Participant 18, 40 years, male, employed)

Table 2 also shows that participants additionally consulted healthcare workers whom they knew personally as well as informal social networks (friends or acquaintances) in their journey. These consults were before or after having sought help from other healthcare providers without any prior solution. This is reflected in the following participant’s comment:

‘[*Name of doctor*] said that he treats only the body, so we must find someone elsewhere. Then we asked [*name of friend*], where can we go for the ear doctor? She said here, she even works at [*name of hospital*].’ (Participant 2, 89 years, female, pensioner)

Participant 11’s wife was frustrated with the cost of healthcare until a friend helped them in this way:

‘We couldn’t afford going to audiologists and so then a friend of ours. This lady, we stay in the same complex. She told us about you.’ (Participant 11, 87 years, male, pensioner)

For participants who sought help from the private healthcare sector, a private doctor, was followed by either a private ENT specialist or a private audiologist or a family or friend or even a combination of these networks, whereas in the public healthcare sector, a PHC consultation was followed by a referral to the ENT before reaching the public audiologist. Evidently, these steps were not linear for everyone. However, results also showed that participants had a preference towards private healthcare rather than using public healthcare.

Data indicated that in most of these long journeys, ENT specialists acted as gatekeepers for reaching audiology services. Participant 15 detailed her journey by stating the following:

‘Yes, last year again, I kept going to the clinic and telling them that I have a hearing problem, then they gave me a referral letter to go to the hospital. I saw the doctor [*specialist*] until he referred me here.’ (Participant 15, 48 years, female, unemployed)

Participant 13 stated:

‘The specialist checked my ears and then he referred me to you.’ (Participant 13, 71 years, male, pensioner)

This is in contrast with the short journeys where participants had immediate access to the ENT specialist.

The researchers consider if participants realised how accessible the ENT Specialist and the audiologist were at the end of their long journeys. Seemingly, more steps were added to participants’ journeys as they sought to resolve their hearing difficulties but were delayed by lack of knowledge of audiology services.

Participant 11 admitted:

‘Well, we didn’t know about it. Oh, I didn’t know there was a possibility that we can come here.’ (Participant 11, 87 years, male, pensioner)

Results indicate that the help-seeking journey of adults with hearing impairment was mostly complex. The process of accessing ear and hearing care specialists was unique for each individual, however, long because of the influence of a variety of environmental factors.

## Discussion

The purpose of this study was to define the help-seeking process from the onset of symptoms to the point of accessing audiology services for adults with hearing impairment.

For most of the participants in this peri-urban community, multiple providers were used to reach ear and hearing care specialists resulting in long journeys, which unfortunately delayed their identification and intervention, thus negatively impacting on their quality of life. The long journeys because of moving from one practitioner to another before reaching the audiologist were also further possibly lengthened as a result of time spent in between these various multiple consultations. Current findings are in line with studies from the same context that mapped the help-seeking journeys of participants with non-auditory medical conditions (Mkize & Uys, 2004; Pronyk et al., 2001). In contrast, short journeys were reported by only a few participants, which is comparable to the current hearing help-seeking research studies as individuals reached audiologists fairly quickly from the time of seeking help. This distinction between short and long journeys is reported in the qualitative investigations conducted on participants’ journeys. (Carson, 2005; Duijvestijn et al., 2003; Laplante-Levesque et al., 2012; Mkize & Uys, 2004). In addition, this difference in findings has been attributed to the predominant social context and the healthcare system in those countries (Laplante-Levesque et al., 2012).

From the current study, it is apparent that adults with hearing impairment did not have immediate access to ear and hearing care services, particularly audiologists. The authors argue that if the ratio of audiologist to patient was more than what has been reported, it is possible that participants could have accessed hearing help sooner. This would have most likely specifically increased other healthcare professionals’ awareness and knowledge of audiologists as a profession, hence improving access. However, the known limited numbers of these professionals negatively influence the knowledge and awareness of other healthcare professionals on the role of audiologists, even in areas, mostly urban areas, where audiologists are available. The unequal distribution of these already limited ear and hearing workforce numbers across the country further negatively impacts health outcomes (Pillay et al., 2020). Studies on mental illness and rheumatoid arthritis in similar contexts also reveal similar findings (Mkize & Uys, 2004; Pati et al., 2019).

With regard to the long journeys, the finding of a variety of formal and informal providers used to access audiologists is consistent with several other studies that reported on this pattern (Moodley et al., 2016; Musinguzi et al., 2018; Pati et al., 2019; Pirani et al., 2015). Though the studies were on other chronic diseases (breast cancer, hypertension, rheumatoid arthritis, Hepatitis C), participants, nevertheless, used a combination of providers to resolve their illnesses. Similar findings have not been reported in the mostly qualitative research studies in HICs on hearing help-seeking (Carson, 2005; Duijvestijn et al., 2003; Knudsen et al., 2010; Laplante-Levesque et al., 2012). The lack of evidence can be ascribed to investigations being conducted in homogenous contexts. However, the common pattern found in other non-auditory conditions validates this study’s finding as investigations were conducted in similar environments; this provides a greater understanding of the contextual factors influencing help-seeking behaviour.

Interestingly, research evidence has indicated that because of the pluralistic nature of the South African healthcare system, adults seeking help tend to use both private and public healthcare in their journeys (Moodley et al., 2016; Pronyk et al., 2001). This resulted in an ongoing complex process with multiple visits to the same healthcare providers. Unfortunately, this finding on selection and choice of multiple healthcare providers has not been reported in hearing help-seeking research (Carson, 2005; Duijvestijn et al., 2003; Knudsen et al., 2010; Laplante-Levesque et al., 2012).

The current study’s finding has, however, been reported by several authors (Moodley et al., 2016; Pati et al., 2019; Pirani et al., 2015; Pronyk et al., 2001). These authors cited healthcare-related reasons including quality of care, inappropriate investigations and a lack of referral as some of the reasons for private and public healthcare usage. Socio-economic conditions (finances) and preference of healthcare provider also influenced these long journeys as reported by participants who sought help first in private but could not continue because of the consultation costs of the private specialists. The current researchers posit that participants had a preferential bias towards the care received from private healthcare professionals, but lifestyle factors (retired; unemployed; cost of care) prevented them from continuing with the same professionals. This is interesting considering that the public healthcare system offers free or low-cost healthcare services for all, especially for the elderly, pregnant women and children under the age of 6 years (Van der Hoeven et al., 2012). Mild symptoms may have also contributed to using lower levels of private healthcare (over-the-counter medicine, private medical doctors) before seeking help from secondary and tertiary levels of healthcare.

Evidently, structural factors in the society as well as inequalities of a developing context impacted access to the ideal caregiver. Although, not implemented yet, current researchers anticipate that universal health coverage under the planned South African Government’s NHI may address these inequalities (Parliament of South Africa, 2019).

Another finding that reflects the multi-cultural context of this current study is the use of family and friends to access audiology services. Word of mouth and knowledge of audiology services played an important role in help-seeking by connecting adults with hearing impairment to the ideal healthcare provider. This is not surprising as more value is placed on the collective versus the individual in diverse contexts (Zhao et al., 2015). In such contexts, help-seeking is part of a community’s identity and is therefore understood from a socio-cultural perspective. Evidence in hearing help-seeking research has not reported on this finding (Carson, 2005; Knudsen et al., 2010; Saunders et al., 2012).

Though reasons for different pathways were not explored, it appears that participants sought help through what was accessible and affordable to them at that time. However, when the hearing difficulties did not resolve, participants continued seeking help from other sources that then contributed to the pattern of long journeys. Implications about the possible lack of outcomes audits in healthcare in this context are raised by this finding. Furthermore, in the context of preventive audiology and the bigger study on ‘hearing help-seeking patterns of adults with hearing impairment’, this study sheds light on the process of accessing ear and hearing care services within this context. Rich perspectives of the interaction of the healthcare user with the healthcare system have been provided.

The study suggests several factors that could contribute to the long journeys in accessing audiology services for adults with hearing impairment in the peri-urban community in South Africa. Limited availability of audiologists and structural inequalities in the healthcare system, with a reported professional-to-patient ratio could result in longer waiting times and delayed access to audiologists, contributing to the overall duration of the help-seeking journey. Additionally, healthcare system disparities, as illustrated by the dual healthcare system introduce disparities in access. The public healthcare sector, where services are often more affordable, may experience resource constraints and longer waiting times. Participants may initially seek help in the public sector but face delays in accessing specialised audiology services. Next, complex referral pathways that participants in the study often had to navigate, involving primary, secondary and tertiary care, with the involvement of ENT specialists as gatekeepers added additional steps to the referral pathway. Complex and convoluted referral processes could contribute to delays in reaching audiologists. Moreover, health illiteracy, with limited awareness or understanding of hearing health services among the participants could contribute to delays. Some participants may not have been aware of the availability of audiology services, leading to missed opportunities for early intervention. Additionally, socio-economic factors such as retirement, unemployment and financial constraints, influenced participants’ choices in seeking healthcare. Participants may have faced financial barriers that affected their ability to access private healthcare services promptly. Lastly, the influence of social networks cannot be ignored. Participants often sought advice from friends, family or acquaintances in their help-seeking process. While social networks can provide valuable support, they may also introduce additional steps in the journey if recommendations lead to multiple consultations before reaching an audiologist. These factors collectively contribute to the complexity and length of the help-seeking journeys observed in the study. Addressing these challenges could potentially streamline the process and facilitate earlier intervention for adults with hearing impairment.

While the study provides valuable insights into the help-seeking process for adults with hearing impairment in a peri-urban community in South Africa, there are some methodological weaknesses that should be considered in the interpretation of the findings. Firstly, the study acknowledges a small sample size (23 participants), which may limit the generalisability of the findings. A larger and more diverse sample could provide a more comprehensive understanding of the help-seeking patterns in the broader South African population. Secondly, there is limited diversity in participants, with the majority of participants in the study being classified as older adults (73.9%), with an average age of 67.8 years. This limits the representation of younger age groups and may not capture the experiences of a more diverse population, including those who might be more actively employed or have different socio-economic backgrounds. Thirdly, the single-centred data collection, where data were collected from a single public hospital in a mostly rural province may not fully capture the variability in help-seeking experiences across different healthcare settings, urban and rural environments and various regions of South Africa. Fourthly, the inclusion and exclusion criteria that focused on participants who had their initial audiology consultation in the department, excluded individuals who sought help through other channels. This could potentially limit the understanding of help-seeking behaviours that do not involve formal audiology departments. Addressing these methodological weaknesses in future research could enhance the robustness and applicability of the study’s findings.

## Conclusion

The study highlights the fact that accessing audiology services for adults with hearing impairment in the peri-urban community in South Africa is a complex process. Participants experienced diverse and often lengthy journeys involving multiple healthcare providers and social networks. Long journeys are common. A significant majority of participants (74%) reported long journeys to access audiology services. These journeys involved interactions with various healthcare providers, both in the public and private sectors, as well as informal social networks. Social, economic and structural factors influence access, with the findings indicating that social-economic factors, health illiteracy and structural challenges in the healthcare system contribute to the prolonged help-seeking journeys. Factors such as retirement, financial constraints and a dual healthcare system with disparities impact how individuals access audiology services. Limited awareness of audiology services, ENT specialists acting as gatekeepers, role of social networks in help-seeking, need for increased awareness and education, impact of healthcare system disparities and implications for Universal Health Coverage (NHI) are all highlighted. The study raises implications for the planned NHI in South Africa, suggesting that universal health coverage could play a vital role in addressing societal inequalities in accessing healthcare, including audiology services. In conclusion, the study underscores the need for targeted interventions to streamline the process of accessing audiology services, considering the unique socio-economic and healthcare system context in the peri-urban community in South Africa.

## Appendix 1

### Interview Schedule

#### Biographical information

Participant code: ______

Gender: Male or Female

DoB: ______________

Ethnicity: ______________

Degree of Hearing loss: ____________

Occupation: Unemployed/ Employed/Self-employed:

Student: Yes or No.

Participant: alone or accompanied by significant other

I would like you to tell me about your journey of seeking help for your hearing difficulties.

1. Tell me about the time when you started noticing that there was a problem with your ear/hearing.
2. Describe the journey that you took or followed from the very first sign/s that made you see that there was a problem (first symptom/s) to the audiologist/reaching this hospital?
  - Probe for pathway or process taken.
  - How did they actually seek help? How was the process (facilitators and barriers)?
